# The Emergence of Embryonic Myosin Heavy Chain during Branchiomeric Muscle Development

**DOI:** 10.3390/life12060785

**Published:** 2022-05-25

**Authors:** Imadeldin Yahya, Marion Böing, Dorit Hockman, Beate Brand-Saberi, Gabriela Morosan-Puopolo

**Affiliations:** 1 Department of Anatomy, Faculty of Veterinary Medicine, University of Khartoum, Khartoum 11115, Sudan; imadeldin.yahya@rub.de; 2 Department of Anatomy and Molecular Embryology, Ruhr University Bochum, 44801 Bochum, Germany; marion.boeing@rub.de (M.B.); beate.brand-saberi@rub.de (B.B.-S.); 3 Division of Cell Biology, Department of Human Biology, Neuroscience Institute, Faculty of Health Sciences, University of Cape Town, Cape Town 7700, South Africa; dorit.hockman@uct.ac.za

**Keywords:** MyHC, mouse embryos, branchiomeric muscles, non-ectomesenchymal crest cells, neck muscles, chicken embryos

## Abstract

A prerequisite for discovering the properties and therapeutic potential of branchiomeric muscles is an understanding of their fate determination, pattering and differentiation. Although the expression of differentiation markers such as myosin heavy chain (MyHC) during trunk myogenesis has been more intensively studied, little is known about its expression in the developing branchiomeric muscle anlagen. To shed light on this, we traced the onset of MyHC expression in the facial and neck muscle anlagen by using the whole-mount in situ hybridization between embryonic days E9.5 and E15.5 in the mouse. Unlike trunk muscle, the facial and neck muscle anlagen express MyHC at late stages. Within the branchiomeric muscles, our results showed variation in the emergence of MyHC expression. MyHC was first detected in the first arch-derived muscle anlagen, while its expression in the second arch-derived muscle and non-somitic neck muscle began at a later time point. Additionally, we show that non-ectomesenchymal neural crest invasion of the second branchial arch is delayed compared with that of the first brachial arch in chicken embryos. Thus, our findings reflect the timing underlying branchiomeric muscle differentiation.

## 1. Introduction

By now, it is widely accepted that branchiomeric and trunk muscles have different developmental histories [1]. Skeletal muscles in the trunk and limbs derive from somites (segmented paraxial mesoderm), whereas the majority of head muscle originates from the unsegmented cranial paraxial mesoderm [2,3,4]. Furthermore, head and trunk muscles have divergent genetic networks that control myogenesis [1,5,6]. The early skeletal muscle progenitor cells originate in the somites, expressing Pax3 followed by Pax7, and undergo long-range migration to generate the tongue, limbs and diaphragm muscles [7]. Pax3 and Pax7 genes keep these progenitor cells in an immature state and activate the myogenic determination genes Myf5 and MyoD [1,4,8,9]. The skeletal muscles of the face, known as branchiomeric muscles, originate from the cranial paraxial mesoderm [10]. Together with cranial neural crest cells (NCCs), cranial paraxial mesodermal cells migrate into the branchial arches [6,10,11]. The first branchial arch mesoderm contributes to the formation of mastication muscles, whereas the second arch mesoderm gives rise to facial expression muscles [2,11,12]. The vertebrate branchiomeric muscles are molecularly, morphologically and evolutionarily distinct from those of the trunk [6]. This view fits well with the concept that their progenitor cells have the ability to form both types of striated (skeletal and cardiac) muscles [1,11,13,14,15,16]. In accordance with these results, immunostaining for the cardiac alpha MyHC revealed its specific presence in the heart and first arch-derived muscles in both humans and rabbits [17]. The branchiomeric muscle progenitor cells do not express the Pax3 gene but instead express Pitx2, Tbx1, Musculin, Islet1 and Nkx2.5 [6,18,19,20,21,22]. Similar to Pax3/7 in the trunk and limbs, these transcription factors were shown to act as upstream regulators of branchiomeric muscle formation [9,10,23,24]. The onset of branchiomeric myogenesis in the first branchial arch is regulated by the homeobox transcription factor Pitx2, which regulates MyoR and Capsulin expression [12]. In mouse embryos lacking Pitx2, the first arch-derived but not second arch-derived muscles fail to develop [25]. Initiation of the branchiomeric myogenesis in the second branchial arch requires Tbx1, which regulates Myf5 and MyoD expression [12,25,26]. Neck muscle progenitors are located in the transition zone between somitic and cranial paraxial mesoderm [6,14,27,28]. Non-somitic neck muscles originate from most caudal branchial arches and develop like head muscles rather than following the myogenic program used by the trunk muscles [14,27]. The onset of myogenic determination varied markedly in skeletal muscles throughout the body. Once myogenic determination genes are activated, myogenic differentiation is thought to happen in a similar manner as in the body [1,29,30,31].

As soon as committed, a large number of myogenic progenitor cells fuse with each other to generate myofibers [7]. In vertebrates, myofibers are generated in two different phases, namely the primary (embryonic) and fetal phases [7,9]. In mice, the embryonic phase (embryonic day E9.5–14.5) takes place to generate primary fibers [7]. Between E14.5 and E17.5, a new wave of myogenesis takes place. This phase is usually referred to as secondary myogenesis and involves the fusion of fetal myoblasts to contribute to the bulk of skeletal muscle fibers present at birth [7,9,32]. The MyHCs are present in different isoforms encoded by different genes [33]. Of the seven mammalian skeletal muscle MyHCs, two developmental isoforms, MyHC embryonic and MyHC perinatal, are expressed during embryonic and fetal phases [7]. A previous study reported that skeletal MyHC transcripts were first noticed in mouse embryos between E9 and E10 in the myotomes of the most rostral somites [34]. The embryonic and perinatal MyHC are transiently expressed during embryonic development and disappear in adult muscle [7,33,35]. However, it has been found that there is a persistent expression of developmental MyHC in specialized skeletal muscles, such as the first arch derived muscle (mastication muscles) and extraocular muscles, during adult phases [33]. Interestingly, these isoforms seem to be transiently re-expressed during skeletal muscle regeneration following injury or disease [7,33,35,36].

Although several studies have reported the expression of MyHC during development [33,34,37], little is known about the MyHC expression in the branchiomeric muscles. It is especially interesting to establish when MyHC expression in these muscles emerges. The aim of this study, therefore, is to establish when MyHC in the mouse branchiomeric muscle cells emerge. We have also investigated the emergence of ectomesenchymal NCCs during the development of chicken first and second branchial arches. Our analysis revealed that MyHC transcripts first become apparent in the first arch-derived muscles. These observations are in accordance with the late invasion of neural crest-derived cells that populate the branchial arches and that have a non-ectomesenchymal fate.

## 2. Materials and Methods

### 2.1. Preparation of Probes for Whole-Mount In Situ Hybridisation

MyHC DIG-labeled RNA probes were synthesized as previously described [38]. Briefly, target sequences of mouse MyHC gene were obtained by RT-PCR using gene-specific primers (Table 1). The short (564 bp) and long (835 bp) RT-PCR products were cloned into the pDrive vector. For the preparation of the antisense RNA probe, the plasmids were linearized with restriction enzymes (Table 1) and synthesized with T7 or SP6 RNA polymerase. The sense RNA probes for MyHC were used as control. The probes were labeled with the digoxigenin-RNA-labeling kit (Roche, Germany). MyoG and Sox10 chicken probes were cloned in our laboratory [28].

### 2.2. Mouse Embryos

Wildtype mice were provided by the Animals Unit, Faculty of Medicine, Ruhr University Bochum. The mice were maintained under specific-pathogen-free conditions in a well-ventilated cage. Mice were mated overnight, and the female mice were checked for the vaginal plug the next day morning. The presence of a vaginal plug was considered as on day 0.5 of pregnancy. Pregnant females were sacrificed in accordance with the German Animal Welfare Act, and the embryos were fixed overnight in 4% PFA at 4 °C. A total of 31 embryos were analyzed (E9.5, E10.5: 3 embryos at each stage; E11.5–E14.5: 4 embryos at each stage; E15.5: 5 embryos).

### 2.3. Whole-Mount In Situ Hybridization

Whole-mount in situ hybridization was performed as described previously [39]. Briefly, mouse and chicken embryos were treated with proteinase K, refixed and hybridized overnight with DIG-labeled antisense probes. RNA probes for mouse MyHC were 835-bp and 564-bp. The hybridization product was visualized by using an anti-digoxigenin antibody fragment conjugated to alkaline phosphatase with respective substrates. Sox10 and MyoG hybridized chicken embryos were further sectioned by using a Leica vibratome at a thickness of 50 μm. Selected sections were processed for immunostaining or embedded in Aquatex from Merck (Darmstadt, Germany).

### 2.4. Immunostaining

Chicken neural crest cells were detected on vibratome sections after in situ hybridization using the HNK1 antibody (Developmental Studies Hybridoma Bank) [38]. Immunostaining on vibratome sections was performed as described in [39].

### 2.5. Microscopy and Imaging

The mouse and chicken embryos were photographed after the whole-mount in situ hybridization by using a Leica MZFLIII microscope and a Leica DC 300F digital camera. The photos were further processed using GIMP software.

## 3. Results

### 3.1. Emergence of MyHC-Expressing Myogenic Cells in Mouse Branchiomeric Muscles

**Figure 1:**
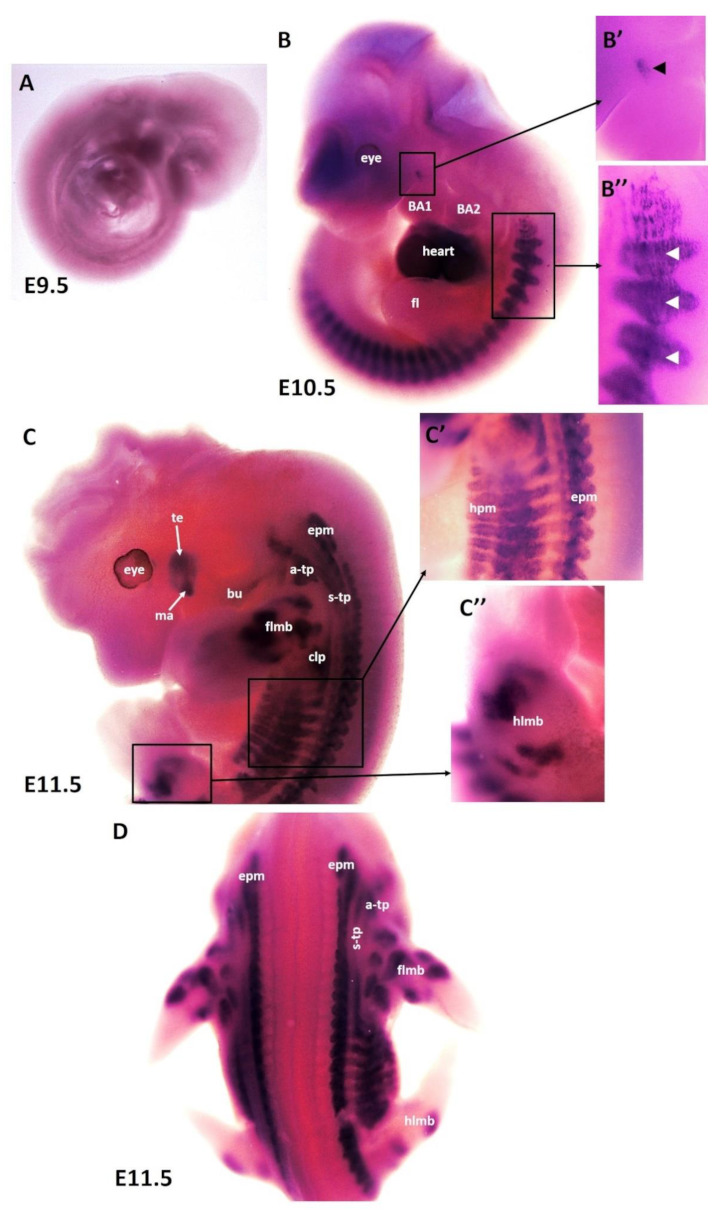
**Expression of MyHC in early-stage mouse embryos.** (**A**–**D**) Whole-mount in situ hybridization for MyHC mRNA expression from E9.5–E11.5 of development. (**B’**,**B”**,**C’**,**C”**) higher magnifications of the areas indicated by the boxes in (**B**,**C**). (**B**) Note labeling of somites (white arrowheads), heart and first arch-derived muscles anlage by MyHC (black arrowhead). (**C**) MyHC is expressed in the first arch-derived muscle (te, ma) and second arch-derived muscle anlagen (bu). MyHC is also expressed in non-somitic neck muscles (a-trap, s-trap) and epaxial and hypaxial muscle anlagen. atp—acromiotrapezius; epm—epaxial musculature; flbm—fore limb muscle anlagen; hlbm—hind limb muscle anlagen; hpm—hypaxial muscle anlagen; mas—masseter; stp—spinotrapezius; te—temporal.

To better understand the patterning and differentiation of branchiomeric muscles, we needed to define the timing underlying their differentiation pattern. Using the whole-mount in situ hybridization, we performed a time course for the expression of MyHC mRNA with emphasis on embryonic days E9.5–E14.5 (embryonic phase). Wildtype mouse embryos (*n* = 31) of different developmental stages were collected for this study. No expression of MyHC was detected at embryonic stage E9.5 (Figure 1A). The earliest expression of MyHC transcripts was observed at embryonic stage E10.5 in the somites (Figure 1B,B”). MyHC was strongly expressed in the developing heart (Figure 1B). A small region of MyHC expression was observed in the first branchial arch (Figure 1B,B’). At E10.5, even though the second branchial arch was well-developed, there was no expression of MyHC in its myogenic mesodermal core (Figure 1B). At E11.5, the expression domain in the somitic mesoderm became more pronounced at E11.5 (Figure 1C). MyHC expression labeled the individual first arch-derived muscles (masseter and temporalis) but, with the exception of the buccinator muscle, was also not expressed in the second arch-derived muscle anlagen (Figure 1C). In the anlagen of the other second arch-derived muscles, MyHC staining did not emerge before embryonic day E12.5. MyHC is also detected in the epaxial musculature, hypaxial musculature and limb muscle anlagen (forelimb and hindlimb) (Figure 1C,D). Non-somitic muscles comprise three different muscle groups, namely trapezius groups (acromio-trapezius and spino-trapezius), sternocleidomastoideus muscles and splenius muscles [14,26,27,28,39]. MyHC was observed in trapezius neck muscle groups (acromio-trapezius and spino-trapezius) (Figure 1C,D).

**Figure 2:**
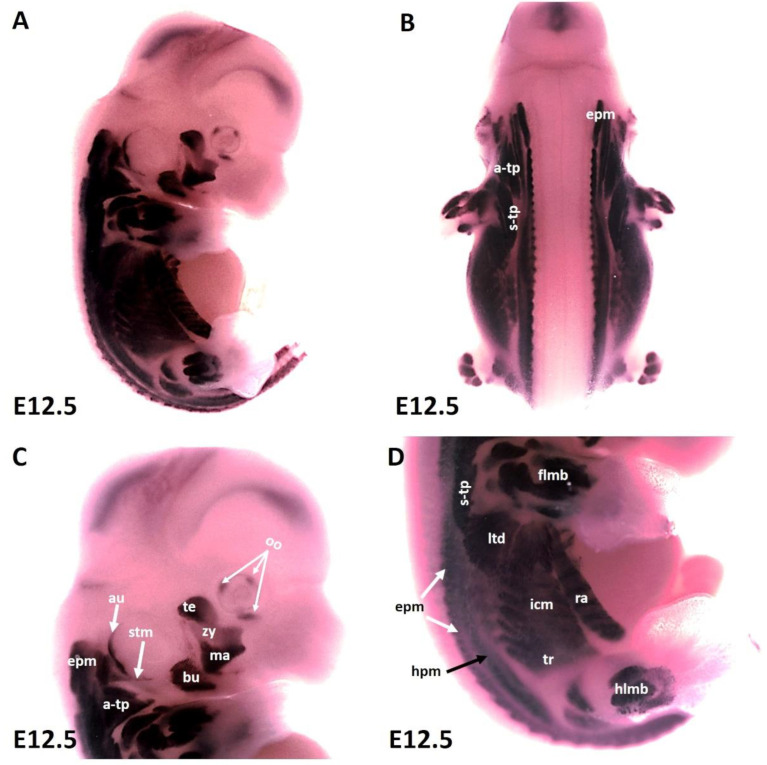
**Expression of MyHC at stage E12.5.** (**A**,**B**) Whole-mount in situ hybridization for MyHC. (**C**,**D**) Higher magnification of the photo in (**A**). (**C**) MyHC transcripts are detected strongly in the first arch-derived muscle anlagen (te, ma), neck muscles (a-trap, s-trap, stm) and second arch-derived muscle anlagen (bu, oo, zy). (**D**) MyHC transcripts are also observed in the thoracic and abdominal (ltd, icm, tr, ra), epaxial, hypaxial, fore limb and hind limb muscle anlagen. atp—acromiotrapezius; au—auricularis; epm—epaxial musculature; flbm—fore limb muscle anlagen; hlbm—hind limb muscle anlagen; hpm—hypaxial muscle anlagen; ltd—latissimus dorsi; mas—masseter; oo—orbicularis oculi; stp—spinotrapezius; stm—sternocleidomastoideus, te—temporalis, zy—zygomaticus.

As development proceeds, the trapezius muscle groups (also known as non-somitic neck muscles) and limbs muscle anlagen (Figure 2A–D) show increasing MyHC expression at E12.5 along with epaxial muscles (Figure 2C,D) first and second arch-derived muscle (Figure 2A,C). We extended our analysis of thoracic and abdominal muscle emergence by analyzing MyHC expression in lateral body wall muscles. MyHC identified individual lateral body wall muscles, such as the intercostals, the transverse abdominus and the rectus abdominus (Figure 2D). MyHC transcripts were also detected in the cutaneous maximus/latissimus dorsi precursors (Figure 2D). In addition, MyHC began to be expressed in the sternocleidomastoideus, auricularis and orbicularis oculi muscles (Figure 2A,C).

**Figure 3:**
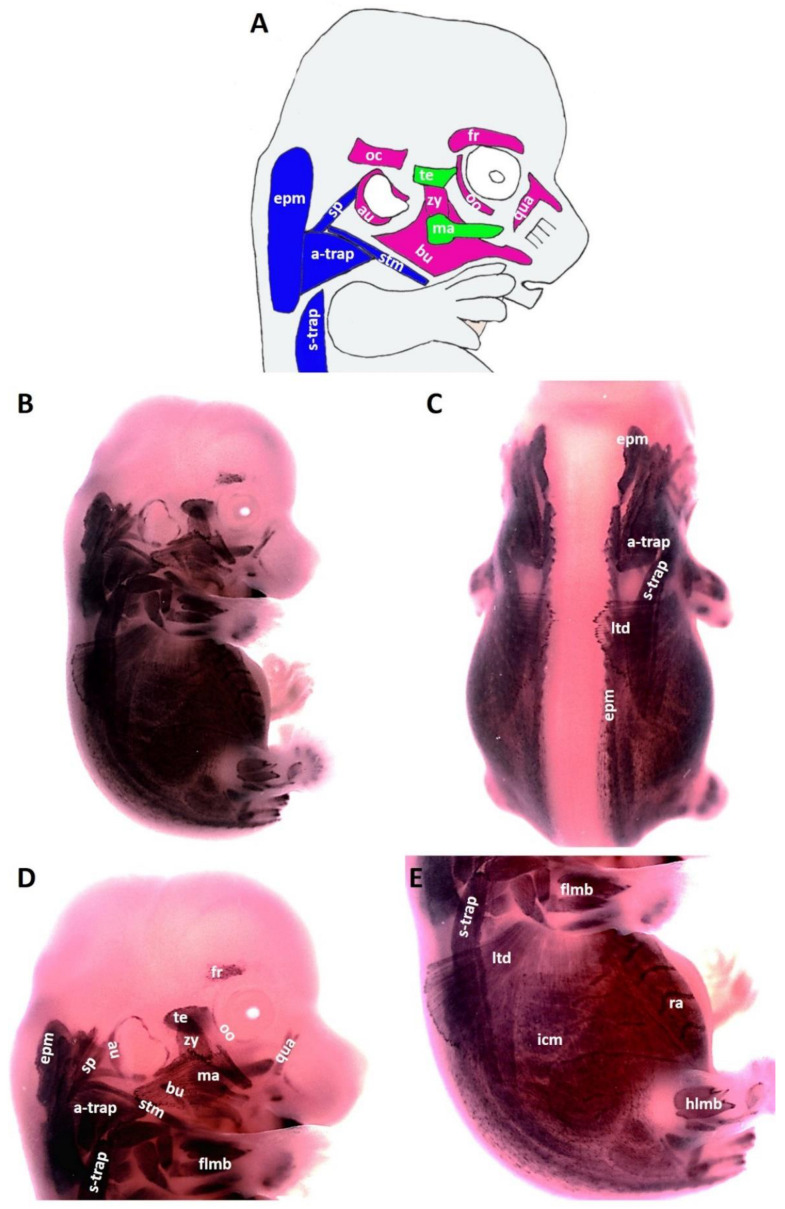
**Expression of MyHC at stage E13.5.** (**A**) Scheme of facial and neck muscles. (**B**,**C**) Whole-mount in situ hybridization for MyHC. (**D**,**E**) Higher magnification of the photo in (**A**). Abbreviations as before and: sp—splenius. (**C**–**E**) Similar to E12.5, MyHC labels the first and second arch-derived muscles, neck muscle, thoracic, abdominal and limb muscle anlagen. (**D**) MyHC expression is first seen at E13.5 in the splenius neck muscle anlagen.

At E13.5, the head of the mouse embryo has grown considerably, leading to the individualization of first and second arch-derived muscles. At this stage E13.5, the following second arch-derived muscles were identifiable: the zygomaticus, the buccinator, the frontalis, the quadratus labii, the orbicularis oculi and the auricularis (Figure 3B,D). MyHC transcripts were also detected in the first arch-derived muscles but stronger than that of stage E12.5 (Figure 3B,D). Strong and lasting expression was detected in the trapezius muscle groups, sternocleidomastoideus, intercostal, transverse abdominus and rectus abdominus muscles. We also observed MyHC expression in splenius muscle (Figure 3B,D). The MyHC expression domain in the lateral body wall muscles is larger than that of stage E12.5 (Figure 3E).

**Figure 4:**
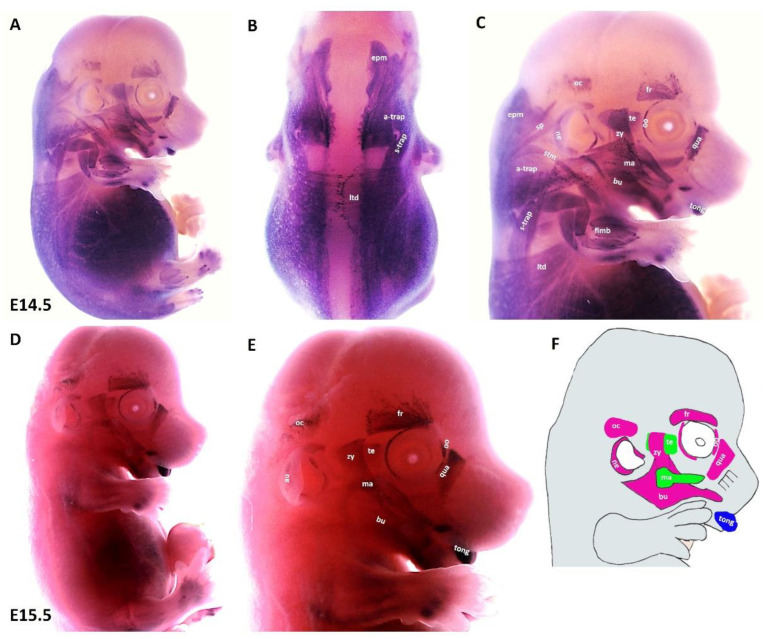
**Expression of MyHC at E14.5 and E15.5.** (**A**,**D**) Whole-mount in situ hybridization for MyHC. (**B**,**C**) Higher magnification of the photo in (**A**). (**E**) Higher magnification of the photo in (**D**). (**F**) Scheme of facial muscles. Abbreviations as before and: oc—occipitalis. (**C**) MyHC is strongly expressed in the first and second arch-derived muscle as well as non-somitic neck muscle groups. (**E**) While second arch-derived muscle anlagen (oc, au, zy, fr, oo, qua and bu) show robust expression, MyHC transcripts are barely detectable in first arch-derived muscle anlagen (te, ma), neck, limbs, thoracic and abdominal muscle anlagen.

We found that during the later stage (E14.5) of development, MyHC expression can be well-detected in the first arch derived muscles (masseter and temporalis), in addition to the second arch-derived muscle anlagen, namely the zygomaticus, the buccinator, the frontalis, the quadratus labii, the orbicularis oculi and the auricularis (Figure 4A,C). Expression associated with occipitals muscle anlagen was first seen at this stage. We also observed strong MyHC expression in non-somitic derived neck muscle anlagen as well as tongue muscle anlagen (Figure 4A–C). In the trunk, MyHC transcripts were also expressed in the lateral body wall, limbs and epaxial neck muscle anlagen (Figure 4A,B). At E15.5, MyHC showed a widespread expression in the myogenic cells that engage with the tongue (Figure 4D,E). Expression in the anlagen of second arch-derived muscles remained higher at this later stage (E15.5) of development, while expression in the first arch-derived muscles was weaker than that of stage E13.5 (Figure 4D,E). Weak expression is also seen in the lateral body wall muscles, limb muscle and neck muscle anlagen (Figure 4D). Taken together, the MyHC is first seen in the first arch-derived muscle anlagen at E10.5, whereas its expression in the second arch-derived muscle anlagen becomes apparent between E12.5 and E13.5 (Table 2).

### 3.2. The Emergence of Non-Ectomesenchymal Neural Crest Cells during Chicken First and Second Arch-Derived Muscle Development

**Figure 5:**
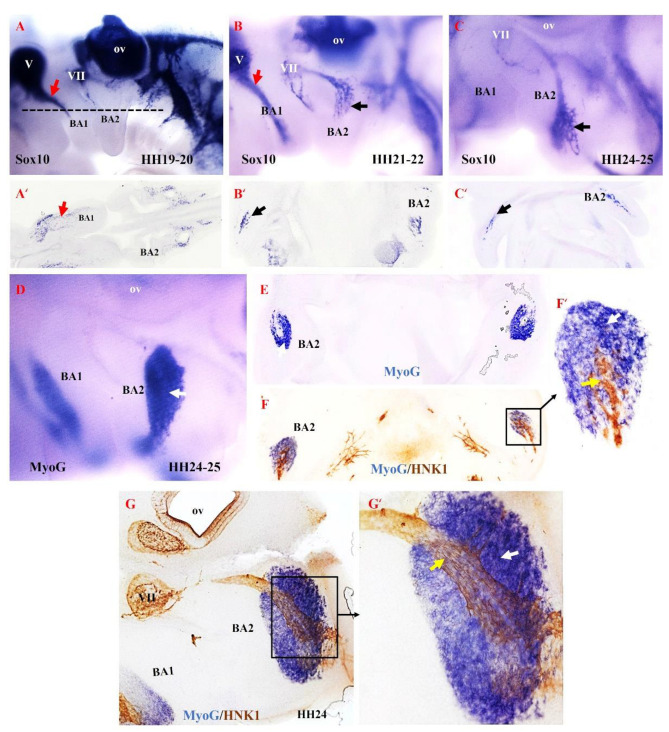
**Relationship between non-ectomesenchymal crest and myogenic cells during development of chicken second branchial arch.** (**A**–**C**) Left lateral views of chicken embryos after whole mount in situ hybridization with Sox10 (black and red arrows denote hybridization signals in the first and second branchial arches). (**A’**–**C’**) Vibratome frontal section of whole-mounted embryos in ((**A**–**C**), indicated by the dotted line in (**A**)). (**D**) Whole-mount in situ hybridization for MyoG. (**E**) Vibratome frontal section of whole-mounted embryos in (**D**). (**F**,**G**) Immunostaining is performed for HNK1 on the same frontal section in (**E**) and sagittal section in (**G**) after whole-mount in situ hybridization. (**F’**,**G’**) Higher magnification of the area indicated by the box in (**F**). MyoG marked the myogenic cells in the second branchial arch (White arrow). The non-ectomesenchymal crest is labeled with HNK1 antibody; yellow arrows point to nerve. BA1—first branchial arch; BA2—second branchial arch; ov—otic vesicle; V—trigeminal ganglion; VII—facial ganglion.

Based on our findings in the mouse model, we hypothesized that the second arch-derived muscles form later during development compared with the first arch-derived muscles. To test our hypothesis in the chicken model, we examined the emergence of NCCs derivatives in the second branchial arch. We analyzed neural crest development since it is thought to be linked to patterning branchiomeric muscle formation [40,41,42,43,44]. NCCs are multipotent progenitor and migratory cell populations that originate from the border between the non-neural ectoderm and the neuroectoderm [38,45,46,47]. Following neural tube closure, NCCs depart from the developing neural tube via an epithelial-to-mesenchymal transition [48]. NCCs then migrate extensively throughout the embryo as a multipotent cell population capable of generating a vast array of derivatives [41,48,49,50]. NCCs can be divided into cranial, cardiac, vagal and trunk NCCs. The cranial NCCs can be grouped into two categories, ectomesenchymal and non-ectomesenchymal. The ectomesenchymal neural crest cells contribute to skeletal muscle elements (connective tissue), whereas the non-ectomesenchymal crest cells give rise to pigment cells, neurons and glia [51,52]. The non-ectomesenchymal cells are characterized by their continued expression of Sox10 at later stages of development [51]. We extended our analysis of mouse MyHC emergence by analyzing Sox10, HNK1 and myogenin (MyoG) expression in brachial arches of chicken embryos (Figure 5). We chose MyoG because it is known to mediate myogenic terminal differentiation [53]. At stages HH19–20, Sox10 expression is observed in the trigeminal ganglion and facial ganglion and extends into the first brachial arch (Figure 5A). At this stage of development, Sox10 is not yet expressed in the second brachial arch. By stages HH21–22 of development, Sox10 expression first becomes apparent in the proximal part of the second branchial arch, whereas Sox10 is largely expressed in the first branchial arch (Figure 5B). At stages HH24–25, Sox10 expression extends into the second branchial arch (Figure 5B). We next compared the expression pattern of Myogenin (MyoG) and HNK1 at stage HH24–25 using a combination of in situ hybridization and immunostaining on vibratome sections. MyoG marked the myogenic cells in the second brachial arch (Figure 5D,E). At this stage, HNK1 likely labels the neural-crest derived cells on the nerve branch targeting the developing muscle cells (Figure 5F,F’,H,H’). Taken together, these results show that the non-ectomesenchymal crest enters the second arch at a later stage compared to the first arch, which corresponds with the later development of the muscle in the second arch.

## 4. Discussion

In all skeletal muscles, the onset of the myogenic program and subsequent differentiation depend on the expression of the myogenic regulatory factor family (Myf5, MyoD, Myogenin and MRF4) [14]. Members of this family have been implicated in the transcriptional control of MyHC [7]. However, upstream regulators of the myogenic regulatory factors differ in different parts of the body [14]. In trunk mesoderm, Pax3 is required to initiate myogenesis [1,4,54]. In the head mesoderm, where Pax3 is not expressed, this role is enacted by a set of transcription factors, including Pitx2, Tbx1, Isl1, MyoR and Capsulin [11,12,14,25,27]. These transcription factors act genetically upstream of myogenic regulatory factors. At E.9.5, Pitx2 was expressed well before the onset of the myogenic program in the first branchial arch mesoderm but not the other branchial arch mesoderm [25]. Myf5 and MyoD mark the onset of myogenic progression, and their expression in the first branchial arch starts between E10 and E10.5 [25]. Tbx1, but not Pitx2, is proposed to be necessary for the initiation of the expression of MyoD and My5 in the second and most caudal branchial mesoderm [12,14,25,39]. 

MyHC is expressed in the myotome of developing somites [34] and in mouse embryoid bodies in vitro [37]. The expression of this gene during the development of facial and neck muscles has been poorly defined. In the present study, using the whole-mount in situ hybridization, we performed a detailed time course for the expression of MyHC in these muscles from embryonic day E9.5 to E15.5 and revealed some interesting observations regarding the onset of its expression. Through the whole-mount in situ hybridization, we identified that the expression of MyHC in branchiomeric muscle anlagen was delayed compared to the limbs and trunk muscle anlagen. In tune with this delay, it has been previously documented that branchiomeric muscles are known to develop late [1,6]. The authors report that the muscle stem cells are laid down later in branchiomeric muscles compared to trunk muscles [1]. Interestingly, the onset of its expression within the developing branchiomeric muscles varies. Expression of MyHC in the first arch-derived muscle anlagen was first detected at E10.5 and became more robust between E12.5–13.5. The expression of mRNA in second arch-derived muscle anlagen was first detected at E11.5 and persisted until E14.5. The difference in the myogenic program for the development of first and second arch-derived muscles has been well documented [25,26,28,30,31]. The transition from progenitor cells state to specified/differentiated cells phase is important for successful muscle formation [55]. A plausible developmental explanation for the delay of MyHC expression is well-matched with findings that cranial neural crest cells regulate myogenesis in the head region by specifically influencing the rate of cell proliferation and differentiation within the branchial arch mesodermal core [41]. Tzahor and his colleagues reported that Wnt signals from the dorsal neural tube induce myogenesis in the trunk mesoderm and block myogenesis in the cranial paraxial mesoderm [56]. In the face, branchiomeric mesoderm derived muscle progenitor cells fuse with each other to generate a myofiber, which is attached to specific cranial neural crest-derived connective tissue (skeletal muscle elements) in a highly coordinated manner [41]. Taken together, the crosstalk between neural crest cells and cranial paraxial mesodermal cells during branchiomeric muscle development regulates the patterning and differentiation of these muscles.

There are approximately 80 skeletal muscles in the human neck [39]. The mammalian neck consists of somite-derived muscle groups (epaxial and hypaxial) and non-somitic (branchiomeric muscles) muscles [3,14,27,28,39]. Skeletal muscle progenitor cells in the branchial arches 3–6 are thought to give rise to non-somitic neck muscles, for example, the trapezius muscle groups and sternocleidomastoideus muscle [14,39]. Through the whole-mount in situ hybridization, we revealed the emergence of MyHC in the somitic and non-somitic neck muscle anlagen between E11.5 and E13.5. Here again, the expression of MyHC in non-somitic neck muscle anlagen was delayed compared to the somite-derived neck muscle and trunk muscles. Using genetically modified mice, Heude and his colleagues outlined new boundaries for neural crest and mesodermal contributions to the skeletal muscle elements in the neck region. Moreover, they defined a unique genetic program for somite-derived neck muscle groups that is distinct from that of trunk muscles [39].

## 5. Conclusions

In conclusion, the emergence of MyHC expression in the branchiomeric muscle anlagen appears to be distinct from that of the trunk muscle anlagen: variation in the timing of development among the different branchiomeric muscle groups is seen.

## Figures and Tables

**Table 1:** Primers for RNA probes of mice MyHC genes.

Probes	Product Length	Enzymes	Promotor/Polymerase	Primers Sequences (5′–3′)
Long	835	XbaI (anti-sense)	T7	F: GTCCTTCCTCAAACCCTTAAAGTA
		XhoI (sense)	SP6	R: TCGTTCCTCACAGTCTTGGC
Short	564	XhoI (anti-sense)	SP6	F: GCAATCAGGAACCTTCGGAACA
		XbaI (sense)	T7	R: CACCTCGTTTTCAAGCTCCC

**Table 2:** Summary of the branchiomeric muscles that expressed MyHC in the mouse embryos from E9.5 to E15.5.

	Branchiomeric Muscles		
Stage	First Arch-Derived Muscles (Mastication Muscles)	Second Arch-Derived Muscles (Facial Expression Muscles)	Caudal Arch-Derived Muscles (Non-Somitic Neck Muscles)
**E9.5**	not detected	not detected	not detected
**E10.5**	mesodermal core	not detected	not detected
**E11.5**	masseter temporalis	buccinator	acromiotrapezius spinotrapezius
**E12.5**	masseter temporalis	buccinator orbicularis oculi zygomaticus auricularis	acromiotrapezius spinotrapezius sternocleidomastoideus
**E13.5**	masseter temporalis	buccinator orbicularis oculi zygomaticus auricularis frontalis quadratus labii	acromiotrapezius spinotrapezius sternocleidomastoideus splenius
**E14.5**	masseter temporalis	buccinator orbicularis oculi zygomaticus auricularis frontalis quadratus labii occipitals	acromiotrapezius spinotrapezius sternocleidomastoideus splenius
**E15.5**	masseter temporalis	buccinator orbicularis oculi zygomaticus auricularis frontalis quadratus labii occipitals	not detected

## Data Availability

Not applicable.
